# Application of a Biomimetic Nanoparticle-Based Mock Virus to Determine SARS-CoV-2 Neutralizing Antibody Levels in Blood Samples Using a Lateral Flow Assay

**DOI:** 10.1021/acs.analchem.3c04372

**Published:** 2024-02-09

**Authors:** Silvia Schobesberger, Helena Thumfart, Florian Selinger, Sarah Spitz, Carla Gonzalez, Lei Pei, Marko Poglitsch, Peter Ertl

**Affiliations:** † TU Wien, Faculty of Technical Chemistry, Getreidemarkt 9, 1060 Vienna, Austria; ‡ Covirabio GmbH, Brehmstraße 14a, 1110 Vienna, Austria

## Abstract

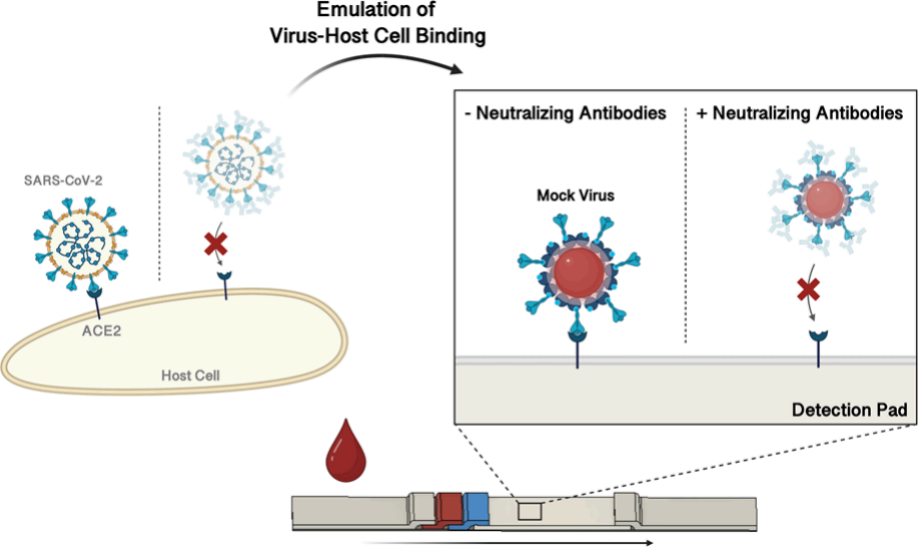

The presence of neutralizing antibodies against SARS-CoV-2 in blood, acquired through previous infection or vaccination, is known to prevent the (re)occurrence of outbreaks unless the virus mutates. Therefore, the measurement of neutralizing antibodies constitutes an indispensable tool in assessing an individual’s and a population’s immunity against SARS-CoV-2. For this reason, we have developed an innovative lateral flow assay (LFA) capable of detecting blood-derived neutralizing antibodies using a biomimetic SARS-CoV-2 mock virus system. Here, functionalized gold nanoparticles (AuNPs) featuring the trimeric spike (S) protein at its surface imitate the virus’s structure and are applied to monitor the presence and efficacy of neutralizing antibodies in blood samples. The detection principle relies on the interaction between mock virus and the immobilized angiotensin-converting enzyme 2 (ACE2) receptor, which is inhibited when neutralizing antibodies are present. To further enhance the sensitivity of our competitive assay and identify low titers of neutralizing antibodies, an additional mixing pad is embedded into the device to increase the interaction time between mock virus and neutralizing antibodies. The developed LFA is benchmarked against the WHO International Standard (21/338) and demonstrated reliable quantification of neutralizing antibodies that inhibit ACE2 binding events down to a detection limit of an antibody titer of 59 IU/mL. Additional validation using whole blood and plasma samples showed reproducible results and good comparability to a laboratory-based reference test, thus highlighting its applicability for point-of-care testing.

The outbreak of SARS-CoV-2 in 2019 caused 768 million infections and 7 million deaths, reported as of July 2023.^1^ To combat the pandemic, vaccines have been developed and globally administered, which has reduced the risk of severe infections by more than 90.8%, hospitalization by 95.4%, and death by 85.8%.^2^ Despite these medical successes, reinfections and disease occurrence are still prominent in all global communities, thus making the assessment of neutralizing antibodies against SARS-CoV-2 a valuable tool to monitor a population’s vaccine- or infection-induced immunity.^3^ Furthermore, neutralizing antibody monitoring is valuable for determining the vaccine’s efficiency, and therefore, several antibody neutralization assays have been developed over the years to assess/detect the presence of antibodies in the bloodstream.^4^ In case of an infection, the entry of SARS-CoV-2 into a host cell is dependent on the initial interaction between the receptor binding domain (RBD) of the spike (S) protein, a homotrimer located on the surface of the virus, and the angiotensin-converting enzyme 2 (ACE2) receptor present on the cell’s surface.^5^ Thus, the standard virus neutralization test mixes replicative SARS-CoV-2 with the sample and investigates its cell entry. However, the assay needs to be performed in a biosafety level 3 (BSL-3) laboratory or, when using a pseudovirus, in a biosafety level 2 (BSL-2) laboratory.^3,4^ These labor- and time-intensive tests require trained personnel, cause high costs, and are inherently difficult to scale up. To overcome the drawbacks of virus-based systems, surrogate neutralization assays based on ELISA technology have emerged, analyzing the inhibition of the protein–protein interactions in the presence of neutralizing antibodies.^6^ Although ELISA technology is amenable to large-scale testing using automated pipetting and read-out systems, increasing the throughput of these methods is still labor-intensive and time-consuming and requires trained personnel since untested blood still needs to be handled in a BSL-2 laboratory. An alternative method is based on lateral flow assays (LFAs), which have been extensively used during the pandemic to detect infections rapidly. Due to their ease of use, low costs, and the ability to implement LFAs in areas where access to RT-qPCR is limited, these systems have become essential for point-of-care diagnostics.^7^ In fact, several studies have already reported the development of a LFA for the detection of neutralizing antibodies against SARS-CoV-2, employing functionalized gold nanoparticles (AuNPs)^8−10^ or other labeling agents such as nanoshells,^11,12^ latex microspheres,^13,14^ quantum dots,^15^ europium nanoparticles,^16^ cellulose nanobeads,^17^ polydopamine nanoparticles,^18^ and aggregation-induced emission luminogen encapsulated polystyrene nanoparticles.^19^ However, these studies mainly rely on neutralizing the ACE2–RBD interaction but do not consider the full-length trimeric S protein, consisting of an S1 subunit with RBD and the S2 subunit (for each monomer). This aspect is essential since neutralizing antibodies are also known to bind alternative epitopes in the N-terminal domain (part of S1) and thus may restrain the S protein’s conformational changes^20^ or epitopes in S2 that prevent the membrane fusion of the virus with the host cell.^21^ Whereas, among others, the S2 subunit epitopes are more conserved, especially RBD is under high selective pressure and readily mutates between variants, thus reducing the effectiveness of neutralizing antibodies targeting this domain.^22^ A recent plaque reduction neutralization assay revealed that the neutralization activity against S1 is almost twice as high as against RBD, suggesting the presence of additional neutralizing antibodies that do not target RBD itself.^23^ Another study reported the plasma’s inability to inhibit the ACE2–RBD interaction while simultaneously showing virus neutralization activity in a pseudovirus neutralization assay.^24^ A comparative analysis of a neutralization assay with fully replicative SARS-CoV-2 and a pseudovirus with S protein on the virus’s surface demonstrated that the neutralization activity mainly depends on the S protein.^25^ Consequently, trimeric S proteins in surrogate neutralization assays may provide more accurate neutralization levels than in currently utilized systems.^26^ Therefore, the full-length trimeric S protein of the targeted SARS-CoV-2 variant mimics the virus’s structure more accurately in a mock virus system.

To address the existing limitations of SARS-CoV-2 immunity testing, our study aims to establish an advanced point-of-care neutralization LFA that mimics the comprehensive interactions involving antibodies, the virus, and the ACE2 receptor. Here, a biomimetic mock virus generated by modifying a AuNP with trimeric S proteins is introduced to detect the presence of neutralizing antibodies and their ability to inhibit ACE2 binding. The nanoparticle simultaneously functions as the mock virus and its label. Although the diameter of a virion is in the range of 60–140 nm,^27−29^ we decided to use 40 nm AuNPs since the streptavidin coating and the additionally added S proteins with a size of 20–25 nm^30−32^ further increase the size of the nanoparticle. AuNPs with a diameter of 40 nm are well-established in LFAs due to advantages like optical visibility by the naked eye and commercial availability. The optical properties are known to change with the nanoparticle size, meaning that the visibility decreases with increasing nanoparticle size. Consequently, the line’s contrast to the membrane decreases, reducing the assay’s sensitivity and read-out ability without expensive equipment. To emulate the three-dimensional structure of SARS-CoV-2, 40 nm AuNPs are modified with trimeric S protein (Figure 1A). These so-called mock virus particles strongly bind to ACE2 in the absence of neutralizing antibodies. In our study, serial dilutions of reference material (e.g., WHO International Standard (21/338)) are used to characterize the developed LFA. This aspect is essential since the standardization of serological methods determining antibody titers is still a major limitation for the comparability of various diagnostic assays.^4,23,33^ To ensure that the test can be performed with blood drawn from a finger prick sample, as shown in Figure 1, the final LFA is developed for a blood volume of 20 μL. In the presence of neutralizing antibodies, the S protein and ACE2 binding is inhibited, and no line appears (Figure 1B). In a small validation study with blood samples, antibody titers are quantified by image analysis and compared to other commercially available antibody tests.

**Figure 1:**
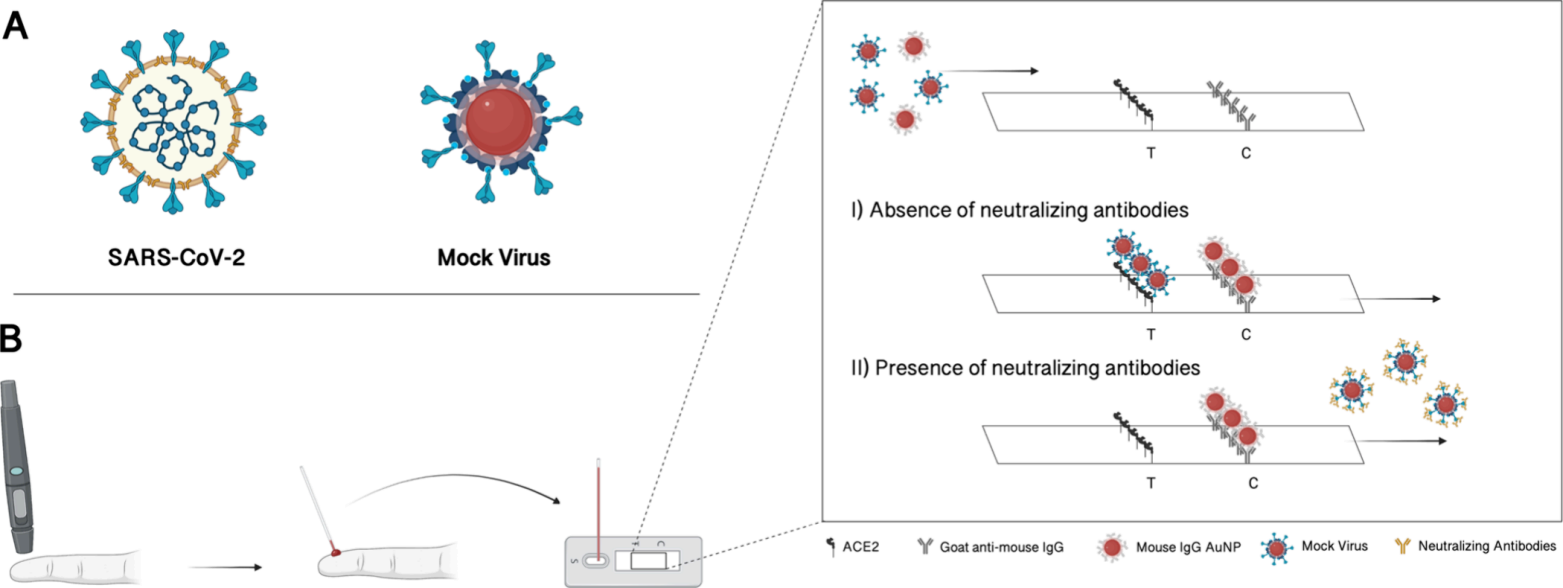
Overview of assay design. (A) Schematic illustration of a SARS-CoV-2 and a mock virus, a AuNP functionalized with S proteins. (B) Blood sampling from a finger prick before the sample is applied to the LFA. (I) In the absence of neutralizing antibodies, the mock virus binds to ACE2 at the test line. (II) In the presence of neutralizing antibodies, the binding of the mock virus to ACE2 is inhibited. T, test line; C, control line; AuNP, gold nanoparticle.

## Experimental Section

### Materials

The WHO International Standard (NIBSC code: 21/338) was obtained from the National Institute for Biological Standards and Control (NIBSC) and the reference material EURM-017 from the Joint Research Centre of the European Commission. Whole blood samples were sourced from the Red Cross Austria and approved by its ethical review committee. Human serum H4522 was procured from Merck.

### Preparation of AuNPs

50 μL of streptavidin modified AuNPs (OD10, Abcam) were conjugated with 1 μg of biotinylated SARS-CoV-2 S trimer proteins (ACROBiosystems). The mixture was incubated for 2 h at room temperature, shaking at 700 rpm using a ThermoCylcer R (Thermo Fisher). After this, free biotin-binding sites were blocked with 900 μM biotin for 15 min, followed by washing the conjugate twice with PBS/Tween 20 (0.05%). For the LFA, AuNP conjugates were stored dry in a conjugate pad using Whatman ST17 (Cytiva). An AuNP conjugate mixture consisting of 11% mouse IgG AuNP conjugates (BBI Solutions) and 89% of the beforehand prepared S protein AuNP conjugates was prepared, centrifuged using a Centrifuge 5424 (Eppendorf), and resuspended in conjugate buffer (PBS/Tween 20(0.05%) with 10% sucrose) to a final OD of 6.3. Each conjugate pad was then dried with 3.5 μL of the prepared AuNP conjugate mixture. Transmission electron microscopy (TEM) of the AuNPs was performed with a FEI TECNAI F20 field emission TEM. Particle size and polydispersity index (PDI) of the AuNPs was determined by ZetaSizer Nano ZSP (Malvern Panalytical Ltd. Instruments).

### Characterization of Functionalized AuNPs

The protocol to determine the electrophoretic mobility was adapted from Fagúndez et al.^34^ Briefly, 10 μL of AuNP solution (OD10) was loaded into an agarose gel (1% w/v in TAE buffer) and run for 5 min at 100 V. The gel was imaged with a Molecular Imager ChemiDoc XRS System (Bio-Rad Laboratories). For the absorbance measurements, the AuNP solutions were diluted and absorbance spectra of the AuNPs were recorded with the plate reader EnSpire 2300 (PerkinElmer).

### LFA Assembly

The nitrocellulose membrane Immunopore RP (Cytiva) was used to immobilize capture reagents. The test and control line of each nitrocellulose strip (3 × 18 mm) consisted of 0.18 μg of ACE2 (Covirabio) and 0.18 μg of goat anti-mouse IgG (Thermo Fisher Scientific) dispensed on the membrane using an AD1520 Aspirate Dispense System (BioDot). After drying the membrane, the test strip was assembled by placing the nitrocellulose membrane on a double-sided adhesive. On one end, the absorbance pad Whatman CF1 (3 × 20 mm) (Cytiva) was added, and on the other end, the conjugate pad Whatman ST17 (3 × 3 mm) (Cytiva) was added. Finally, the blood separation sample pad (3 × 6 or 3 × 25 mm) (Senova Immunoassay Systems) was placed, overlapping the conjugate pad. For the final LFA assembly, a mixing pad (3 × 4 mm) was placed between the nitrocellulose membrane and conjugate pad, using the blood separation sample pad.

### LFA Testing

To obtain pure plasma, 2 mL of blood from an EDTA blood collection tube was transferred into a microcentrifuge tube (Eppendorf) and centrifuged for 15 min at 2500*g*. The supernatant was transferred to another microcentrifuge tube (Eppendorf) and centrifuged again for 10 min at 2500*g*. Pure plasma was collected and tested on the lateral flow test strip. Dilutions of the WHO International Standard were prepared with Diluent (Roche). The lateral flow strips were tested with 12 μL plasma/serum or 20 μL of blood, followed by the addition of 30 μL of Hanks’ Balanced Salt Solution (Merck). After 15 min, the assay was completed. Then, images of the dry strips were taken with a Gel Reader (Bio-Rad Laboratories) and analyzed using the open-source software FIJI. The blood samples were additionally analyzed with Elecsys Anti-SARS-CoV-2 S (Roche) and Gmate COVID-19 Neutralizing antibody (Philosys), performed as stated by the vendors.

## Results and Discussion

### Establishment and Characterization of the Mock Virus

**Figure 2:**
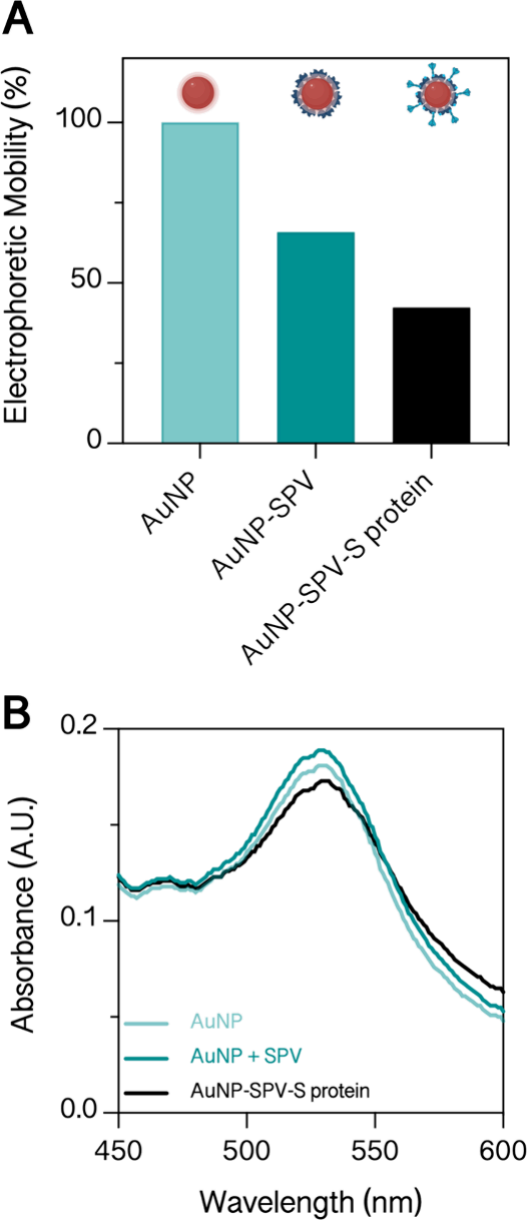
Characterization of bare AuNP, AuNP coated with streptavidin (AuNP-SPV), and the mock virus (AuNP-SPV-S protein). (A) Electrophoretic mobility decreases with functionalization. (B) Absorbance spectrum changes when AuNP-SPV is functionalized with S protein.

For the development of a mock virus, the three-dimensional orientation and complete architecture of the surface protein are vital for mimicking the virus’s structure. Thus, to control the direction of the S protein on the nanoparticle’s surface, site-specific biotinylation of S protein was used to functionalize streptavidin-coated AuNPs, minimizing the interference of labeling with protein structure and function. In addition, the orientation of the S proteins on AuNPs becomes highly reproducible. The biotinylated S proteins were conjugated with streptavidin-coated AuNPs, and the functionalization was subsequently analyzed by comparing the electrophoretic mobility of bare AuNPs, AuNPs coated with streptavidin (AuNP-SPV) and mock viruses (AuNP-SPV-S protein). The conjugation of proteins reduced the electrophoretic mobility of the AuNPs by 34% when coated with streptavidin and by 58% when additionally functionalized with S protein (Figures 2A and S1). The observed decrease in electrophoretic mobility confirmed the successful conjugation of the mock virus system for the LFA. Additionally, the AuNP properties were characterized by comparing the absorbance spectrum of bare AuNPs and functionalized AuNPs, demonstrating a slight shift of the absorbance spectrum at wavelengths above 530 nm due to the conjugation of proteins, especially for AuNP-SPV-S protein (Figure 2B). This was further supported by measuring the hydrodynamic diameter, determining a size of 44.1 ± 1.6 nm for bare AuNPs, 90.8 ± 1.0 nm for AuNP-SPV, and 110.6 ± 1.3 nm for AuNP-SPV-S protein. Thus, the size of the mock virus is comparable to SARS-CoV-2. However, TEM images (Figure S2) showed that the AuNPs still have a size of ∼40 nm, which is also supported by the absorbance peak at 530 nm. Therefore, the size increase from AuNP to AuNP-SPV is based on the surface functionalization protocol of the manufacturer, whereas the increase from AuNP-SPV to AuNP-SPV-S protein can be explained by the size of the S protein (20–25 nm^30−32^). The polydispersity index (PDI) of AuNP-SPV-S protein was measured to be 0.16, indicating a monodispersed nanoparticle suspension. Thereby, we confirmed the successful functionalization of AuNP-SPV with S protein, resulting in a mock virus with a comparable size to SARS-CoV-2.

### Development of the LFA Test Strip

**Figure 3:**
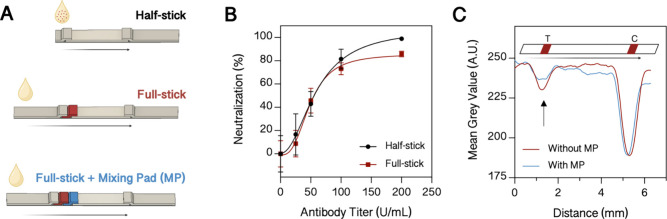
LFA test strip development. (A) Schematic illustration of different tested membrane configurations starting with a half-stick and manual AuNP addition, followed by a full-stick with AuNPs stored in the integrated conjugate pad. The final configuration comprises a full-stick with an integrated mixing pad. (B) Response curve of half-stick and full-stick setups to a serial dilution of EURM-017 (*n* = 3). (C) Integrating the mixing pad into the full-stick configuration decreases the test line intensity and thus increases neutralization (199 U/mL). MP, mixing pad; T, test line; C, control line.

To establish a mock virus-based portable diagnostic system, a three-step workflow was used during the LFA development with increasing LFA configuration complexity: (1) half-stick, (2) full-stick, and (3) full-stick with mixing pad (Figure 3A). First, a half-stick consisting of absorbance, detection, and conjugate pad was used during the early development phase to reduce the number of variable parameters. Mock virus particles and the sample were premixed and then applied onto the conjugate pad, facilitating the assessment of varying AuNP quantities to ensure optimal visualization of the control and test line (Table S1). A serial dilution of EURM-017 serum, a reference material for immunoassays or virus neutralization assays provided by the European Commission, with an antibody titer of 199 U/mL (determined with Elecsys Anti-SARS-CoV-2 S) was employed to appraise the assay’s ability to measure diverse antibody titers. Subsequently, the test strips were imaged and analyzed by quantifying line intensities. Results in Figure 3B show a sigmoidal saturation curve characteristic of a competitive assay setup, where the line intensity decreases with an increasing antibody titer, thus indicating higher neutralization levels. In the next step, a full-stick configuration consisting of absorbance, detection, conjugate, and sample pad, wherein the mock virus is stored within the conjugate pad (as shown in Figure 3A), was employed. Following the application of reference serum, the mock virus particles are released and enter the detection pad. A comparative analysis between the full-stick and the half-stick systems (see black and red traces in Figure 3B) revealed comparable response curves. However, the neutralization of the highest antibody titer (199 U/mL) is significantly lower in the full-stick setup, which indicates reduced interaction or inefficient mixing. To overcome these shortcomings, an additional pad was introduced to increase the interaction time and improve sensitivity.^35^Figure 3C shows that the test line (T) intensity of 199 U/mL was reduced by 15% in the presence of a 4 mm long mixing pad between the conjugate and detection pad. This points to enhanced neutralization, whereas the intensity of the control line (C) remains the same. Another essential aspect to consider is the mixing pad’s material since the pad’s properties affect the retention time. We decided to use the same material as used for the sample pad since it retains red blood cells and consequently prevents potential blood cell leakage into the nitrocellulose membrane. With the integration of a mixing pad, interaction times are increased while simultaneously leaked red blood cells are retained, thus improving assay reliability by the enhanced interaction and the effective elimination of unwanted discoloration (red blood cells) of the detection pad.

### Validation of LFA with WHO International Standard and Blood Samples

**Figure 4:**
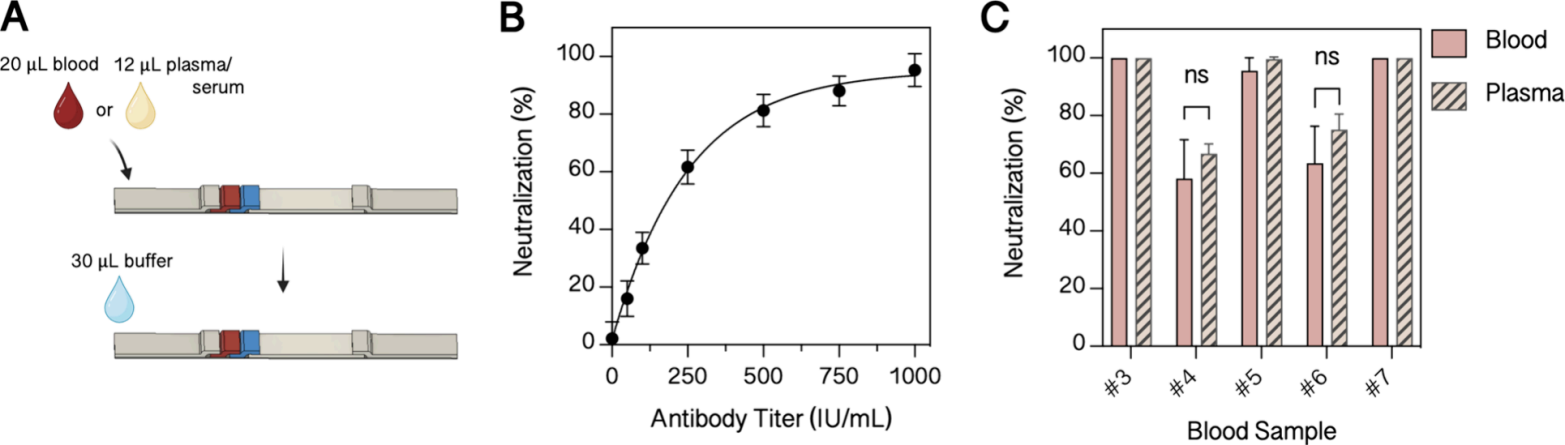
Testing the final LFA configuration. (A) Schematic illustration of the testing procedure. (B) Response curve to serial dilution of the WHO International Standard (21/338) (*n* = 4). (C) Comparison of blood and plasma samples from 5 individuals. Statistical significance by the Welch *t* test: 0.12 (ns), 0.033 (*), 0.002 (**), <0.001 (***) (*n* = 3).

In order to test whole blood, the sample pad’s size was increased from 6 to 25 mm to ensure complete retention of all the red blood cells present in a 20 μL whole blood or 12 μL plasma sample (considering a hematocrit of 40%), while the rinsing buffer volume was simultaneously increased from 15 to 30 μL accordingly (depicted in Figure 4A). A performance validation for the developed LFA was conducted using the WHO International Standard (21/338) to ensure comparability to other testing systems. The WHO reference plasma contains antibodies against different SARS-CoV-2 variants of concern and provides its potency, respectively. However, since the AuNPs were modified with α-S protein, the plasma’s potency against the α-variant (5584 IU/mL) was used as a reference. A serial dilution of the WHO reference plasma was tested, and line intensities were assessed. Results shown in Figure 4B exhibited a detection limit of an antibody titer of 59 IU/mL, a linear range from 0 to 250 IU/mL, and a saturation of antibody concentrations above 500 IU/mL. To cover a broad spectrum of applications, the LFA should be functional with both blood and plasma/serum samples. To confirm that the assumption of a hematocrit of 40% is valid and consequently 12 μL of plasma and 20 μL of blood lead to comparable results, the blood and plasma of five individuals were analyzed. The results of this study are depicted in Figure 4, showing that no significant differences in line intensities between plasma and blood samples are observed, thus pointing to similar assay performance. These results clearly demonstrate that the developed LFA reliably detects neutralizing antibodies from blood and plasma/serum samples. To further demonstrate the applicability of the LFA to detect lower neutralization titers next to the commercially available sera EURM-017 (199 U/mL) and H4522 (0.9 BAU/mL), samples #4, #5, and #6 were further diluted prior to analysis (see Figure S9).

**Figure 5:**
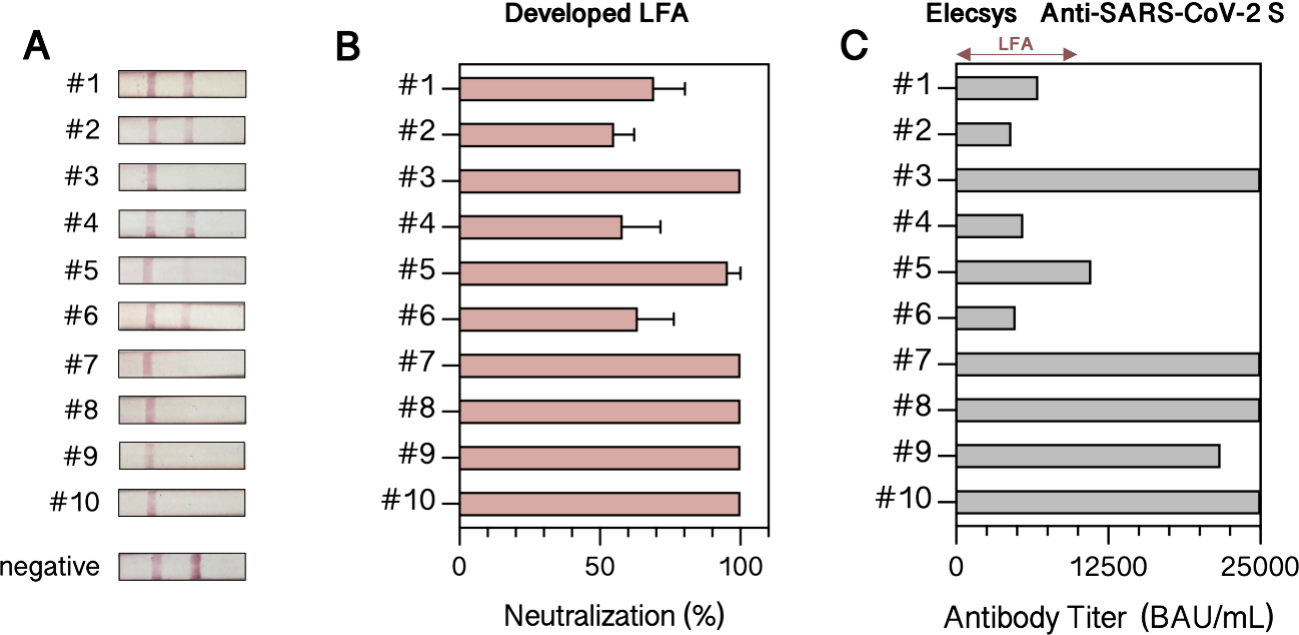
Validation of the developed LFA test strip. (A) Images and (B) analyzed neutralization levels of 10 blood samples tested with the developed LFA (*n* = 3). (C) Antibody titer of blood samples determined by Roche Elecsys Anti-SARS-CoV-2 S.

In a final set of experiments, blood samples from ten individuals are used to benchmark the performance of the mock virus-based LFA against the immunoassay Elecsys Anti-SARS-CoV-2 S and a commercially available LFA. Results shown in Figure 5A revealed no test line for 5 blood samples (see samples #3, #7,#, #8, #9, and #10 in Figure 5A) in the developed LFA, which means antibody titers exceed the threshold of 1000 IU/mL. These results indicate the presence of high titers of neutralizing antibodies against SARS-CoV-2, while donors #1, #2, #4, and #6 show lower levels of neutralizing antibodies between 50% and 80% (see Figure 5B and Table S2). The same ten blood samples were simultaneously analyzed by an external laboratory with the Elecsys Anti-SARS-CoV-2 S test, a serological immunoassay that measures antibodies against RBD but does not test for neutralization. Results are shown in Figure 5C, featuring a broader detection range but a similar pattern. Comparable to our developed LFA, blood samples with antibody titers lower than 1000 IU/mL (samples #1, #2, #4, #6) are identified. Overall, the antibody titers determined by the developed LFA are approximately 20-fold (samples #1, #2, #4, #6, Table S2) lower than those measured by Elecsys Anti-SARS-CoV-2 S, underlining the importance of employing the same reference material in the future since different antibody tests are characterized with different reference materials, especially due to the lack of reference sera at the beginning of the pandemic. As an example, when measuring an antibody titer of 50 IU/mL of the WHO International Standard with Elecsys Anti-SARS-CoV-2 S, an antibody titer of 497 BAU/mL is determined, indicating a 10-fold discrepancy between the plasma’s potency against the α-variant stated by the data sheet (21/338) and the measured value. In addition, when the LFA is performed with AuNP-RBD instead of the mock virus, neutralization is higher for samples #1, #2, and #4 (Figure S7). For sample #5, a 10-fold lower antibody titer was determined (compared to 20-fold lower titers for the other samples) and the LFA using AuNP-RBD shows 100% neutralization compared to the LFA using the mock virus (Figure S8). These apparent discrepancies demonstrate that the neutralization effect could differ when using our developed mock virus system, because a trimeric S protein is used in our LFA while Elecsys Anti-SARS-CoV-2 S, in turn, employs RBD and does not test for neutralization. To study the observed differences further, the mock virus is intended to be employed in more sensitive neutralization tests. Overall, sample #5 further indicates that the maximal detectable antibody titer of our LFA is around 11 000 BAU/mL in terms of Elecsys Anti-SARS-CoV-2 S. Furthermore, in addition to a serological immunoassay, the same blood samples were analyzed using a commercially available LFA that detects neutralizing antibodies inhibiting the binding of RBD to ACE2. Results in Figure S6 show only a faint line for samples #2 and #6, the samples with the lowest antibody titer according to Elecsys Anti-SARS-CoV-2 S, indicating that the commercially available LFA has a lower optical cutoff when compared to the developed LFA utilizing the mock virus (Figure 5A). Overall, characterizing the developed LFA with the WHO International Standard (21/338) enables comparability with other testing methods. Furthermore, the developed LFA demonstrates applicability to both blood and plasma/serum samples and, additionally, enables quantification of neutralizing antibodies.

## Conclusion

The spread of any viral infection and the extent of disease severity is strongly influenced by the immunity prevalent within the population, acquired through either infection or vaccination, which plays a pivotal role in maintaining low hospitalization rates. Particularly, immune protection of vulnerable groups, including elderly, sick, and immunocompromised people, against SARS-CoV-2 (or other viruses) needs to be acquired and/or preserved to eliminate the risk of severe COVID-19 outcomes. Consequently, diagnostic assays capable of detecting the presence of neutralizing antibodies for an increasing number of viruses are needed to identify individuals of high, medium, and low infection risk.

In this study, we propose the use of a mock virus-based rapid diagnostic system that mimics the viral surface structure of SARS-CoV-2 by functionalizing AuNPs with trimeric S protein. The established mock virus is ideally suited for LFAs because the nanogold allows an optical read-out and quantitation of neutralization events that prevent the mock virus from binding to the cell surface receptor ACE2. The test can be performed from blood or plasma/serum and requires only a sample volume of 20 or 12 μL, respectively, which makes it ideally suitable for point-of-care testing. The standard LFA setup was adapted by the additional integration of a mixing pad sandwiched between the conjugate and the detection pad resulting (i) in the efficient removal of all red blood cells that eliminates visual background in the detection zone and (ii) improved interaction times between the mock virus and the neutralizing antibodies leading to a detection limit of an antibody titer of 59 IU/mL. It has to be noted that a direct comparison with available test kits remains challenging due to the absence of standardized reference materials at the beginning of the pandemic. We have addressed this issue by using an international standard material to characterize our developed LFA with the aim of ensuring better comparability to other assays currently under development. This aspect is important for the development of next-generation diagnostic systems and aids in the comparability, harmonization, and standardization in the future.

Furthermore, since the correlation between neutralizing antibody levels and immunity of a person is not fully understood yet, and consequently, analytical cutoff values are currently not defined, rapid diagnostic assays may represent an indispensable tool to collect more data and knowledge. This also means that, as soon as protective titers are determined, point-of-care tests, such as our quantitative LFAs, will significantly contribute to monitoring the population’s immunity and help to understand when revaccination is required. However, it is important to note that one of the limitations is reduced neutralization when the virus mutates. Especially when vaccine-induced neutralization is studied, the S protein of the vaccine strain needs to be implemented into the mock virus. Thus, the LFA needs to be adapted continuously or, to better understand the neutralizing antibodies’ ability to inhibit the ACE2 interaction with the S protein of different variants, an advanced multiplexed diagnostic device using a panel of different mock virus systems may help to identify susceptibilities to different variants. A broader public acceptance and trust in accurate point-of-care diagnostic devices exhibiting adequate sensitivities, robustness, and reliability will ultimately help to control disease outbreaks in the future.

## Supporting Information Available

Additional results of LFA development, including adjustment of line intensity and sample pad length; images of agarose gel and lateral flow test strips; calculated and measured antibody titer of tested samples determined by developed LFA and Elecsys Anti-SARS-CoV-2 S (PDF)

## References

[ref1] WHO Coronavirus (COVID-19) Dashboard|WHO Coronavirus (COVID-19) Dashboard With Vaccination Data; https://covid19.who.int/ (accessed 2023-07-13).

[ref2] Yang Z. R.; Jiang Y. W.; Li F. X.; Liu D.; Lin T. F.; Zhao Z. Y.; Wei C.; Jin Q. Y.; Li X. M.; Jia Y. X.; Zhu F. C.; Yang Z. Y.; Sha F.; Feng Z. J.; Tang J. L. Efficacy of SARS-CoV-2 Vaccines and the Dose–Response Relationship with Three Major Antibodies: A Systematic Review and Meta-Analysis of Randomised Controlled Trials. Lancet Microbe 2023, 4 (4), e236–e246. 10.1016/S2666-5247(22)00390-1. 36868258 PMC9974155

[ref3] Gattinger P.; Ohradanova-Repic A.; Valenta R. Importance, Applications and Features of Assays Measuring SARS-CoV-2 Neutralizing Antibodies. Int. J. Mol. Sci. 2023, 24 (6), 5352 10.3390/ijms24065352. 36982424 PMC10048970

[ref4] Liu K.-T.; Han Y.-J.; Wu G.-H.; Huang K.-Y. A.; Huang P.-N. Overview of Neutralization Assays and International Standard for Detecting SARS-CoV-2 Neutralizing Antibody. Viruses 2022, 14 (7), 1560 10.3390/v14071560. 35891540 PMC9322699

[ref5] Yang J.; Petitjean S. J. L.; Koehler M.; Zhang Q.; Dumitru A. C.; Chen W.; Derclaye S.; Vincent S. P.; Soumillion P.; Alsteens D. Molecular Interaction and Inhibition of SARS-CoV-2 Binding to the ACE2 Receptor. Nat. Commun. 2020, 11 (1), 4541 10.1038/s41467-020-18319-6. 32917884 PMC7486399

[ref6] Tan C. W.; Chia W. N.; Qin X.; Liu P.; Chen M. I. C.; Tiu C.; Hu Z.; Chen V. C. W.; Young B. E.; Sia W. R.; Tan Y. J.; Foo R.; Yi Y.; Lye D. C.; Anderson D. E.; Wang L. F. A SARS-CoV-2 Surrogate Virus Neutralization Test Based on Antibody-Mediated Blockage of ACE2–Spike Protein–Protein Interaction. Nat. Biotechnol. 2020, 38 (9), 1073–1078. 10.1038/s41587-020-0631-z. 32704169

[ref7] Zhou Y.; Wu Y.; Ding L.; Huang X.; Xiong Y. Point-of-Care COVID-19 Diagnostics Powered by Lateral Flow Assay. TrAC Trends in Analytical Chemistry 2021, 145, 116452 10.1016/j.trac.2021.116452. 34629572 PMC8487324

[ref8] Liu Z.; Liang J.; Hu H.; Wu M.; Ma J.; Ma Z.; Ji J.; Chen H.; Li X.; Wang Z.; Luo Y. Development of an Effective Neutralizing Antibody Assay for SARS-CoV-2 Diagnosis. Int. J. Nanomed 2023, 18, 3125–3139. 10.2147/IJN.S408921. PMC10275375 37333734

[ref9] Connelly G. G.; Kirkland O. O.; Bohannon S.; Lim D. C.; Wilson R. M.; Richards E. J.; Tay D. M.; Jee H.; Hellinger R. D.; Hoang N. K.; Hao L.; Chhabra A.; Martin-Alonso C.; Tan E. K. W.; Koehler A. N.; Yaffe M. B.; London W. B.; Lee P. Y.; Krammer F.; Bohannon R. C.; Bhatia S. N.; Sikes H. D.; Li H. Direct Capture of Neutralized RBD Enables Rapid Point-of-Care Assessment of SARS-CoV-2 Neutralizing Antibody Titer. Cell Rep. Methods 2022, 2 (8), 100273 10.1016/j.crmeth.2022.100273. 35942328 PMC9350670

[ref10] Fulford T. S.; Van H.; Gherardin N. A.; Zheng S.; Ciula M.; Drummer H. E.; Redmond S.; Tan H. X.; Boo I.; Center R. J.; Li F.; Grimley S. L.; Wines B. D.; Nguyen T. H. O.; Mordant F. L.; Ellenberg P.; Rowntree L. C.; Kedzierski L.; Cheng A. C.; Doolan D. L.; Matthews G.; Bond K.; Hogarth P. M.; McQuilten Z.; Subbarao K.; Kedzierska K.; Juno J. A.; Wheatley A. K.; Kent S. J.; Williamson D. A.; Purcell D. F. J.; Anderson D. A.; Godfrey D. I. A Point-of-Care Lateral Flow Assay for Neutralising Antibodies against SARS-CoV-2. EBioMedicine 2021, 74, 103729 10.1016/j.ebiom.2021.103729. 34871960 PMC8641961

[ref11] Zhao T.; Liang P.; Ren J.; Zhu J.; Yang X.; Bian H.; Li J.; Cui X.; Fu C.; Xing J.; Wen C.; Zeng J. Gold-Silver Alloy Hollow Nanoshells-Based Lateral Flow Immunoassay for Colorimetric, Photothermal, and SERS Tri-Mode Detection of SARS-CoV-2 Neutralizing Antibody. Anal. Chim. Acta 2023, 1255, 341102 10.1016/j.aca.2023.341102. 37032051 PMC10026621

[ref12] Lake D. F.; Roeder A. J.; Kaleta E.; Jasbi P.; Pfeffer K.; Koelbela C.; Periasamy S.; Kuzmina N.; Bukreyev A.; Grys T. E.; Wu L.; Mills J. R.; McAulay K.; Gonzalez-Moa M.; Seit-Nebi A.; Svarovsky S. Development of a Rapid Point-of-Care Test That Measures Neutralizing Antibodies to SARS-CoV-2. J. Clin Virol 2021, 145, 105024 10.1016/j.jcv.2021.105024. 34781240 PMC8567411

[ref13] Liang Z.; Peng T.; Jiao X.; Zhao Y.; Xie J.; Jiang Y.; Meng B.; Fang X.; Yu X.; Dai X. Latex Microsphere-Based Bicolor Immunochromatography for Qualitative Detection of Neutralizing Antibody against SARS-CoV-2. Biosensors 2022, 12 (2), 103 10.3390/bios12020103. 35200362 PMC8869495

[ref14] Zhang Y.; Chen Y.; He Y.; Li Y.; Zhang X.; Liang J.; He J.; Lu S.; Gao Z.; Xu J.; Tang Y. Development of Receptor Binding Domain-Based Double-Antigen Sandwich Lateral Flow Immunoassay for the Detection and Evaluation of SARS-CoV-2 Neutralizing Antibody in Clinical Sera Samples Compared with the Conventional Virus Neutralization Test. Talanta 2023, 255, 124200 10.1016/j.talanta.2022.124200. 36565525 PMC9767659

[ref15] Li J.; Liu B.; Tang X.; Wu Z.; Lu J.; Liang C.; Hou S.; Zhang L.; Li T.; Zhao W.; Fu Y.; Ke Y.; Li C. Development of a Smartphone-Based Quantum Dot Lateral Flow Immunoassay Strip for Ultrasensitive Detection of Anti-SARS-CoV-2 IgG and Neutralizing Antibodies. Int. J. Infect Dis 2022, 121, 58–65. 10.1016/j.ijid.2022.04.042. 35483554 PMC9040449

[ref16] Duan X.; Shi Y.; Zhang X.; Ge X.; Fan R.; Guo J.; Li Y.; Li G.; Ding Y.; Osman R. A.; Jiang W.; Sun J.; Luan X.; Zhang G. Dual-Detection Fluorescent Immunochromatographic Assay for Quantitative Detection of SARS-CoV-2 Spike RBD-ACE2 Blocking Neutralizing Antibody. Biosens Bioelectron 2022, 199, 113883 10.1016/j.bios.2021.113883. 34942543 PMC8673933

[ref17] Lee J. H.; Lee Y.; Lee S. K.; Kim J.; Lee C. S.; Kim N. H.; Kim H. G. Versatile Role of ACE2-Based Biosensors for Detection of SARS-CoV-2 Variants and Neutralizing Antibodies. Biosens Bioelectron 2022, 203, 114034 10.1016/j.bios.2022.114034. 35114464 PMC8800143

[ref18] Tong H.; Cao C.; You M.; Han S.; Liu Z.; Xiao Y.; He W.; Liu C.; Peng P.; Xue Z.; Gong Y.; Yao C.; Xu F. Artificial Intelligence-Assisted Colorimetric Lateral Flow Immunoassay for Sensitive and Quantitative Detection of COVID-19 Neutralizing Antibody. Biosens Bioelectron 2022, 213, 114449 10.1016/j.bios.2022.114449. 35696869 PMC9174064

[ref19] Bian L.; Li Z.; He A.; Wu B.; Yang H.; Wu Y.; Hu F.; Lin G.; Zhang D. Ultrabright Nanoparticle-Labeled Lateral Flow Immunoassay for Detection of Anti-SARS-CoV-2 Neutralizing Antibodies in Human Serum. Biomaterials 2022, 288, 121694 10.1016/j.biomaterials.2022.121694. 35977850 PMC9360774

[ref20] Chi X.; Yan R.; Zhang J.; Zhang G.; Zhang Y.; Hao M.; Zhang Z.; Fan P.; Dong Y.; Yang Y.; Chen Z.; Guo Y.; Zhang J.; Li Y.; Song X.; Chen Y.; Xia L.; Fu L.; Hou L.; Xu J.; Yu C.; Li J.; Zhou Q.; Chen W. A Neutralizing Human Antibody Binds to the N-Terminal Domain of the Spike Protein of SARS-CoV-2. Science 2020, 369 (6504), 650–655. 10.1126/science.abc6952. 32571838 PMC7319273

[ref21] Pinto D.; Sauer M. M.; Czudnochowski N.; Low J. S.; Alejandra Tortorici M.; Housley M. P.; Noack J.; Walls A. C.; Bowen J. E.; Guarino B.; Rosen L. E.; di Iulio J.; Jerak J.; Kaiser H.; Islam S.; Jaconi S.; Sprugasci N.; Culap K.; Abdelnabi R.; Foo C.; Coelmont L.; Bartha I.; Bianchi S.; Silacci-Fregni C.; Bassi J.; Marzi R.; Vetti E.; Cassotta A.; Ceschi A.; Ferrari P.; Cippà P. E.; Giannini O.; Ceruti S.; Garzoni C.; Riva A.; Benigni F.; Cameroni E.; Piccoli L.; Pizzuto M. S.; Smithey M.; Hong D.; Telenti A.; Lempp F. A.; Neyts J.; Havenar-Daughton C.; Lanzavecchia A.; Sallusto F.; Snell G.; Virgin H. W.; Beltramello M.; Corti D.; Veesler D. Broad Betacoronavirus Neutralization by a Stem Helix–Specific Human Antibody. Science 2021, 373 (6559), 1109–1116. 10.1126/science.abj3321. 34344823 PMC9268357

[ref22] Chen Y.; Zhao X.; Zhou H.; Zhu H.; Jiang S.; Wang P. Broadly Neutralizing Antibodies to SARS-CoV-2 and Other Human Coronaviruses. Nat. Rev. Immunol 2023, 23 (3), 189–199. 10.1038/s41577-022-00784-3. 36168054 PMC9514166

[ref23] Freeman J.; Olson K.; Conklin J.; Shalhoub V.; Johnson B. A.; Bopp N. E.; Fernandez D.; Menachery V. D.; Aguilar P. V. Analytical Characterization of the SARS-CoV-2 EURM-017 Reference Material. Clin Biochem 2022, 101, 19–25. 10.1016/j.clinbiochem.2021.12.009. 34933006 PMC8684092

[ref24] Byazrova M.; Gattinger P.; Astakhova E.; Hofer G.; Khaitov M.; Filatov A.; Valenta R. Dissection of Antibody Responses of Gam-COVID-Vac-Vaccinated Subjects Suggests Involvement of Epitopes Outside RBD in SARS-CoV-2 Neutralization. Int. J. Mol. Sci. 2023, 24 (6), 5104 10.3390/ijms24065104. 36982183 PMC10049224

[ref25] Trinité B.; Tarrés-Freixas F.; Rodon J.; Pradenas E.; Urrea V.; Marfil S.; Rodríguez de la Concepción M. L.; Ávila-Nieto C.; Aguilar-Gurrieri C.; Barajas A.; Ortiz R.; Paredes R.; Mateu L.; Valencia A.; Guallar V.; Ruiz L.; Grau E.; Massanella M.; Puig J.; Chamorro A.; Izquierdo-Useros N.; Segalés J.; Clotet B.; Carrillo J.; Vergara-Alert J.; Blanco J. SARS-CoV-2 Infection Elicits a Rapid Neutralizing Antibody Response That Correlates with Disease Severity. Sci. Rep 2021, 11 (1), 2608 10.1038/s41598-021-81862-9. 33510275 PMC7843981

[ref26] Kim S. J.; Yao Z.; Marsh M. C.; Eckert D. M.; Kay M. S.; Lyakisheva A.; Pasic M.; Bansal A.; Birnboim C.; Jha P.; Galipeau Y.; Langlois M. A.; Delgado J. C.; Elgort M. G.; Campbell R. A.; Middleton E. A.; Stagljar I.; Owen S. C. Homogeneous Surrogate Virus Neutralization Assay to Rapidly Assess Neutralization Activity of Anti-SARS-CoV-2 Antibodies. Nat. Commun. 2022, 13 (1), 3716 10.1038/s41467-022-31300-9. 35778399 PMC9249905

[ref27] Ke Z.; Oton J.; Qu K.; Cortese M.; Zila V.; McKeane L.; Nakane T.; Zivanov J.; Neufeldt C. J.; Cerikan B.; Lu J. M.; Peukes J.; Xiong X.; Kräusslich H. G.; Scheres S. H. W.; Bartenschlager R.; Briggs J. A. G. Structures and Distributions of SARS-CoV-2 Spike Proteins on Intact Virions. Nature 2020, 588 (7838), 498–502. 10.1038/s41586-020-2665-2. 32805734 PMC7116492

[ref28] Perez-Bermejo J. A.; Kang S.; Rockwood S. J.; Simoneau C. R.; Joy D. A.; Silva A. C.; Ramadoss G. N.; Flanigan W. R.; Fozouni P.; Li H.; Chen P.-Y.; Nakamura K.; Whitman J. D.; Hanson P. J.; McManus B. M.; Ott M.; Conklin B. R.; McDevitt T. C. SARS-CoV-2 Infection of Human IPSC Derived Cardiac Cells Reflects Cytopathic Features in Hearts of Patients with COVID-19. Sci. Transl Med. 2021, 13 (590), 7872 10.1126/scitranslmed.abf7872. PMC8128284 33723017

[ref29] Werion A.; Belkhir L.; Perrot M.; Schmit G.; Aydin S.; Chen Z.; Penaloza A.; De Greef J.; Yildiz H.; Pothen L.; Yombi J. C.; Dewulf J.; Scohy A.; Gérard L.; Wittebole X.; Laterre P. F.; Miller S. E.; Devuyst O.; Jadoul M.; Morelle J.; Aboubakar F.; Acid S.; Amini N.; Bailly S.; Beauloye C.; Castanares-Zapatero D.; Coche E.; Collienne C.; Cornette P.; De Brauwer I.; Dechamps M.; Dupriez F.; Froidure A.; Garnir Q.; Gerber B.; Ghaye B.; Gilard I.; Gohy S.; Grégoire C.; Hantson P.; Jacquet L. M.; Kabamba B.; Kautbally S.; Lanthier N.; Larbaoui F.; Liistro G.; Maes F.; Montiel V.; Mwenge B.; Pierard S.; Pilette C.; Pouleur A. C.; Sogorb A.; Starkel P.; Rodriguez-Villalobos H.; Thoma M.; Van Caeneghem O.; Vancraeynest D. SARS-CoV-2 Causes a Specific Dysfunction of the Kidney Proximal Tubule. Kidney Int. 2020, 98 (5), 1296–1307. 10.1016/j.kint.2020.07.019. 32791255 PMC7416689

[ref30] Laue M.; Kauter A.; Hoffmann T.; Möller L.; Michel J.; Nitsche A. Morphometry of SARS-CoV and SARS-CoV-2 Particles in Ultrathin Plastic Sections of Infected Vero Cell Cultures. Sci. Rep 2021, 11 (1), 1–11. 10.1038/s41598-021-82852-7. 33568700 PMC7876034

[ref31] Klein S.; Cortese M.; Winter S. L.; Wachsmuth-Melm M.; Neufeldt C. J.; Cerikan B.; Stanifer M. L.; Boulant S.; Bartenschlager R.; Chlanda P. SARS-CoV-2 Structure and Replication Characterized by in Situ Cryo-Electron Tomography. Nat. Commun. 2020, 11 (1), 5885 10.1038/s41467-020-19619-7. 33208793 PMC7676268

[ref32] Turoňová B.; Sikora M.; Schürmann C.; Hagen W. J. H.; Welsch S.; Blanc F. E. C.; von Bülow S.; Gecht M.; Bagola K.; Hörner C.; van Zandbergen G.; Landry J.; de Azevedo N. T. D.; Mosalaganti S.; Schwarz A.; Covino R.; Mühlebach M. D.; Hummer G.; Locker J. K.; Beck M. In Situ Structural Analysis of SARS-CoV-2 Spike Reveals Flexibility Mediated by Three Hinges. Science 2020, 370 (6513), 203–208. 10.1126/science.abd5223. 32817270 PMC7665311

[ref33] Müller L.; Kannenberg J.; Biemann R.; Hönemann M.; Ackermann G.; Jassoy C. Comparison of the Measured Values of Quantitative SARS-CoV-2 Spike Antibody Assays. J. Clin Virol 2022, 155, 105269 10.1016/j.jcv.2022.105269. 36029637 PMC9388276

[ref34] Fagúndez P.; Botasini S.; Tosar J. P.; Méndez E. Systematic Process Evaluation of the Conjugation of Proteins to Gold Nanoparticles. Heliyon 2021, 7 (6), e07392 10.1016/j.heliyon.2021.e07392. 34307927 PMC8258641

[ref35] Tsai T. T.; Huang T. H.; Chen C. A.; Ho N. Y. J.; Chou Y. J.; Chen C. F. Development a Stacking Pad Design for Enhancing the Sensitivity of Lateral Flow Immunoassay. Sci. Rep 2018, 8 (1), 17319 10.1038/s41598-018-35694-9. 30470789 PMC6251899

