# Supplementary material for: Application of a Biomimetic Nanoparticle-Based Mock Virus to Determine SARS-CoV-2 Neutralizing Antibody Levels in Blood Samples Using a Lateral Flow Assay
Source: Anal Chem. 2024 Feb 9;96(7):2900–7. doi: 10.1021/acs.analchem.3c04372 (PMC10882572; doi:10.1021/acs.analchem.3c04372)
Supplement: Supplementary file 1 — ac3c04372_si_001.pdf [file ac3c04372_si_001.pdf]

Supplementary Information

Application of a Biomimetic Nanoparticle-Based Mock Virus to Determine SARS-CoV-2 Neutralizing Antibody Levels in Blood Samples Using a Lateral Flow Assay

Silvia Schobesberger[1], Helena Thumfart[1], Florian Selinger[1], Sarah Spitz[1], Carla Gonzalez[2], Lei Pei[2], Marko Poglitsch[2] and Peter Ertl[*1]

1. TU Wien, Faculty of Technical Chemistry, Getreidemarkt 9, 1060 Vienna, Austria
2. Covirabio GmbH, Brehmstraße 14a, 1110 Vienna, Austria

*Corresponding author: Peter Ertl, peter.ertl@tuwien.ac.at

Abbreviations: AuNP, gold nanoparticle(s); SPV, streptavidin; S, spike; RBD, receptor binding domain; LFA, lateral flow assay;

Table of Content

Figure S1: Electrophoretic mobility of AuNP
Figure S2: Transmission electron microscopy images of AuNP
Figure S3: Integration of mixing pad
Figure S4: Example of images used for quantification using FIJI
Figure S5: Comparison of 6 and 25 mm long sample pad by testing a serial dilution of WHO International Standard (21/338)
Figure S6: Testing of samples with commercially available LFA detecting neutralizing antibodies.
Figure S7: Comparison of RBD and S protein
Figure S8: Sample #5 tested with LFA utilizing mock virus or AuNP-RBD
Figure S9: Testing of lower antibody titer

Table S1: AuNP mixtures analyzed regarding the intensity ratio between test (T) and control (C) line.
Table S2: Antibody titer determined by developed LFA and Elecsys® Anti-SARS-CoV-2 S

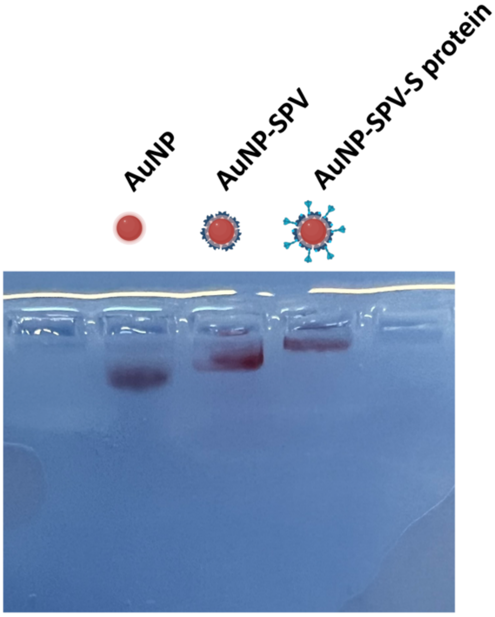

Figure S1: Electrophoretic mobility of AuNP - bare AuNP, AuNP coated with streptavidin (AuNP-SPV) and AuNP-SPV functionalized with trimeric S protein (AuNP-SPV-S protein)

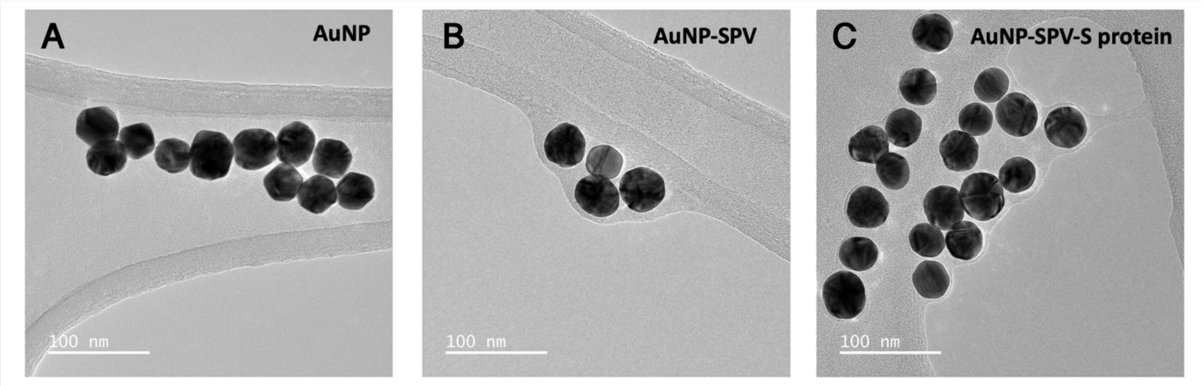

Figure S2: Transmission electron microscopy images of AuNP. A) AuNP, B) AuNP-SPV and C) AuNP-SPV-S protein.

Table S1: AuNP mixtures analyzed regarding the intensity ratio between test (T) and control (C) line. The control line binds mouse IgG AuNP and the test line binds the mock virus, consisting of AuNP functionalized with trimeric S protein.

Mix	Mouse IgG AuNP (%)	Mock Virus (%)	T/C intensity ratio
#1	32	68	0.97 ± 0.01
#2	27	73	0.97 ± 0.01
#3	36	64	1.00 ± 0.05
#4	30	70	0.96 ± 0.03
#5	34	66	1.01 ± 0.01

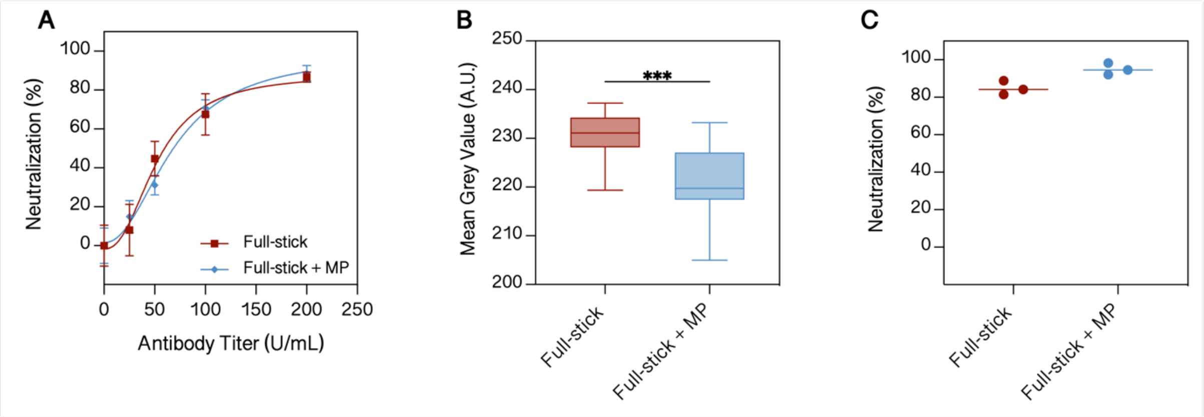

Figure S3: Integration of mixing pad (MP). A) Comparison of full-stick and full-stick with MP. B) Background signal of full-stick and full-stick + MP indicates that the full-stick + MP has a higher background due to the increased length of the strip. C) Neutralization of 199 U/mL with full-stick and full-stick + MP after increasing the volume of buffer from 10 to 15 µl.

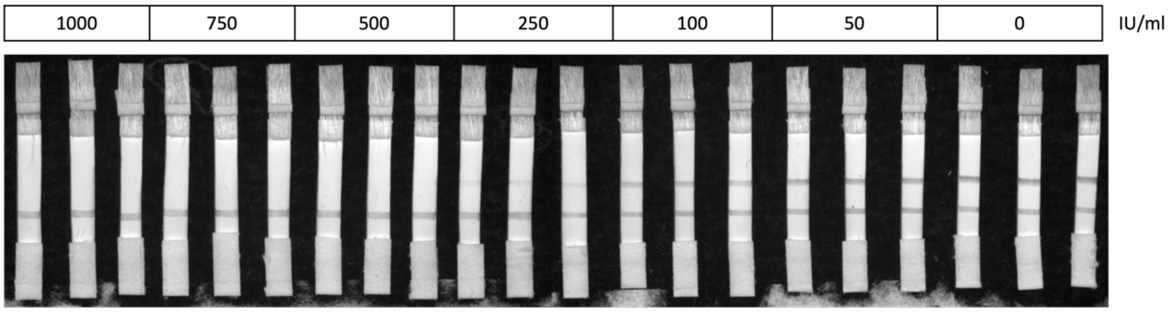

Figure S4: Example of images used for quantification using FIJI.

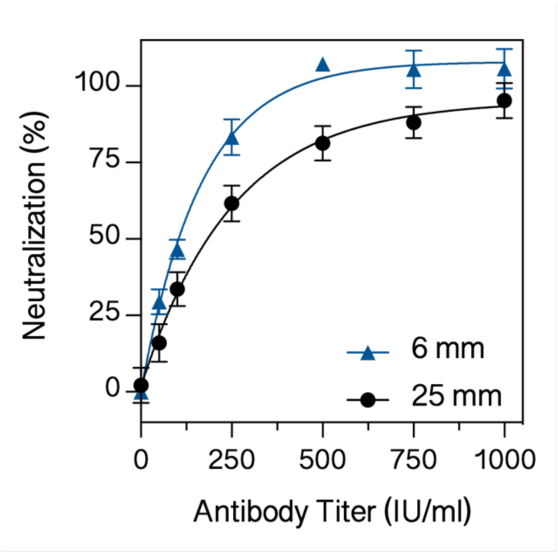

Figure S5: Comparison of 6 and 25 mm long sample pad by testing a serial dilution of WHO International Standard (21/338).

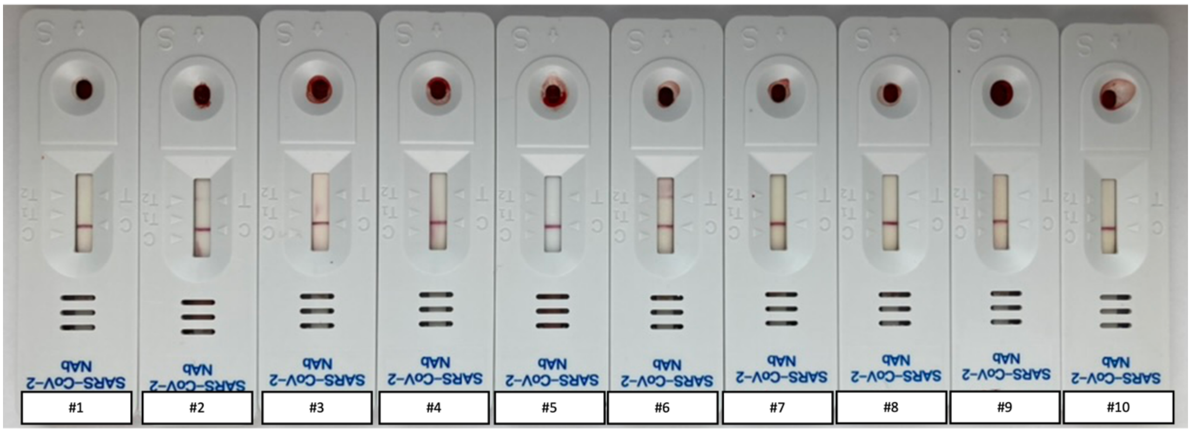

Figure S6: Testing of samples with commercially available LFA detecting neutralizing antibodies.

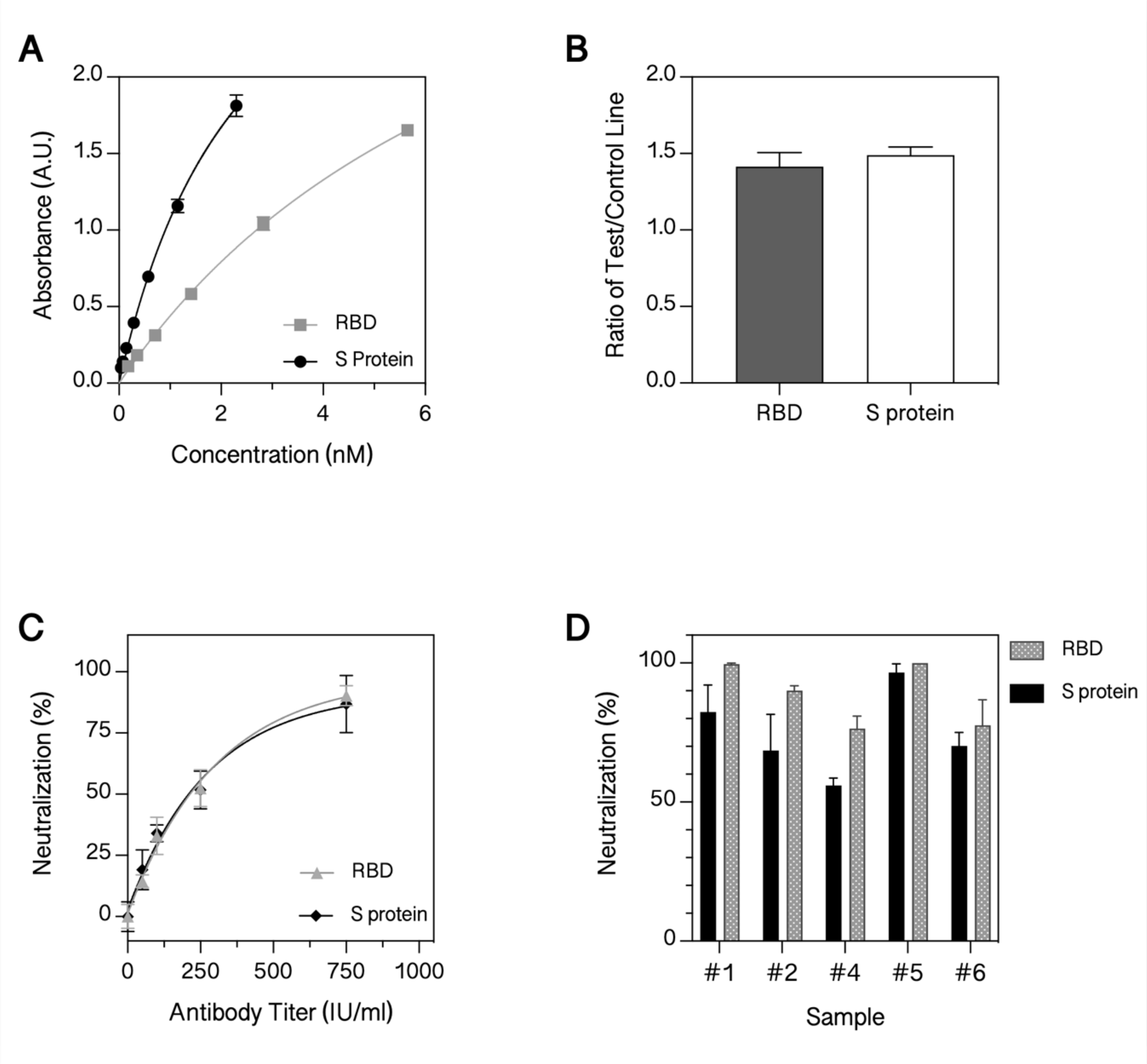

Figure S7: Comparison of RBD and S protein. A) ELISA to study the binding to immobilized ACE2; B) AuNP modified with RBD or S protein tested in LFA; C) Serial dilution of WHO International Standard (21/338) in LFA utilizing trimeric S protein (mock virus)- or RBD-modified AuNP. D) Plasma samples tested with AuNP-RBD and AuNP-SPV-S protein.

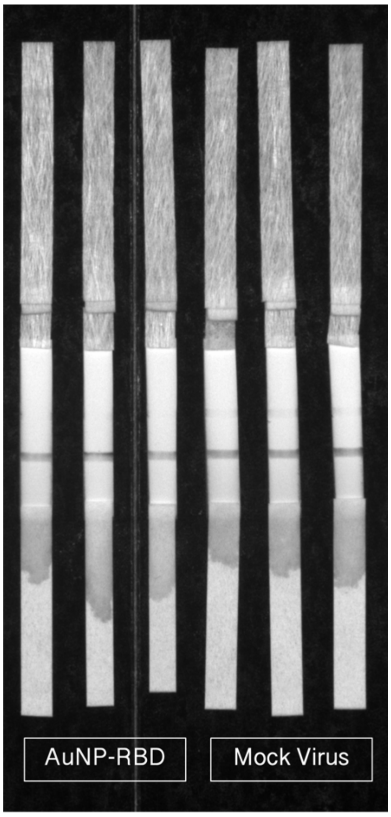

Figure S8: Sample #5 tested with LFA utilizing mock virus or AuNP-RBD.

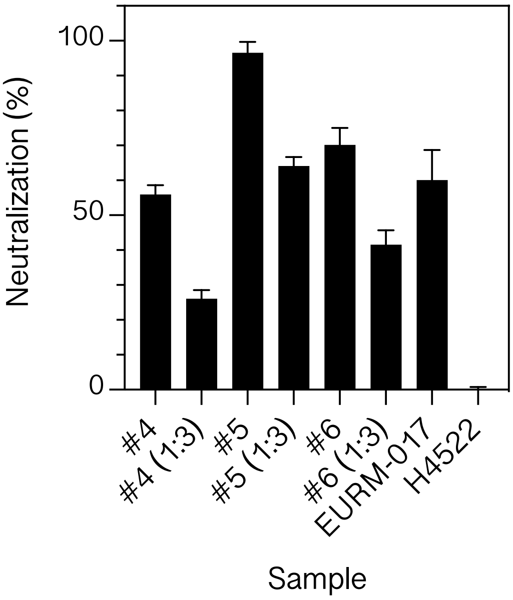

Figure S9: Testing of lower antibody titer. Samples #4, #5 and #6 were diluted 3-fold and compared to the undiluted sample. EURM-017 (199 U/mL stated by vendor) and H4522 (0.9 BAU/mL determined by Elecsys® Anti-SARS-CoV-2 S) are commercially available serum samples.

*Table S2: Antibody titer determined by developed LFA and Elecsys® Anti-SARS-CoV-2 S (*close to the threshold 1000 IU/ml).*

**Sample**	**LFA Antibody Titer (IU/ml)**	**Elecsys® Anti-SARS-CoV-2 S Antibody Titer (BAU/ml)**
#1	323	6756
#2	212	4545
#3	>1000	>25000
#4	233	5501
#5	~1000*	11122
#6	272	4887
#7	>1000	>25000
#8	>1000	>25000
#9	>1000	21724
#10	>1000	>25000
